# A Rare Case of Tissierella praeacuta Bacteremia in the Context of Lower Extremity Cellulitis

**DOI:** 10.7759/cureus.74843

**Published:** 2024-11-30

**Authors:** Gopal Kumar, Sahar Iqbal, Fnu Raja, Thessicar Antoine-Reid

**Affiliations:** 1 Pathology, MetroHealth Medical Center, Cleveland, USA; 2 Pathology, MetroHealth Medical Center, Case Western Reserve University, Cleveland, USA

**Keywords:** gram-variable, lower extremity cellulitis, matrix-assisted laser desorption/ionization-time of flight, severe sepsis, tissierella praeacuta

## Abstract

*Tissierella praeacuta *(*T. praeacuta*)is a gram-variable obligate anaerobe. In this case report, we describe the first documented case of *T. praeacuta* bacteremia in a patient with sepsis resulting from lower extremity cellulitis without concomitant osteomyelitis. During the inpatient course, the patient was treated with IV vancomycin, cefepime, and ertapenem, in addition to surgical debridement and incision and drainage of his foot wound. The patient was discharged to a skilled care nursing facility on ertapenem with significant clinical improvement.

## Introduction

*Tissierella praeacuta* was first described as a gram-variable, non-spore-forming, obligate anaerobe, and rod-shaped bacterium discovered in 1908 by P. H. Tissier [1,2]. It is found commonly in the human gastrointestinal tract and environmental sources such as soil [3]. It is a rare cause of infection in humans, with very few documented case reports in medical literature involving brain abscess [4], hepatic abscess, pyonephrosis [2], pyometra [5], chronic sacral wounds, decubitus ulcers [3], pseudoarthrosis of the femur, osteomyelitis [1], and gas gangrene of the eyelid [6]. Identifying *T. praeacuta *can be challenging, as conventional methods are widely used in most labs. It may require newer technology, such as matrix-assisted laser desorption-ionization-time of flight (MALDI-TOF) and 16s RNA sequencing. In this case report, we describe the first documented case of *T. praeacuta* bacteremia in a patient with sepsis resulting from lower extremity cellulitis, confirmed by MALDI-TOF.

## Case presentation

A 78-year-old male former smoker with a past medical history of chronic obstructive pulmonary disease, hypertension, diet-controlled type 2 diabetes mellitus, and peripheral arterial disease complicated by right above-knee amputation presented to the emergency department for dizziness aggravated by positional changes. He reported no history of vision change, headache, recent fall, or trauma. He denied having shortness of breath, chest pain, fever, chills, dysuria, hematuria, or drug abuse. His vital signs were notable for mild tachycardia. The labs were remarkable for leukocytosis with a white cell count of (>25.5 109/L) with left shift and elevated high sensitivity troponin, erythrocyte sedimentation rate (ESR), and C-reactive protein (CPR).

**Figure 1:**
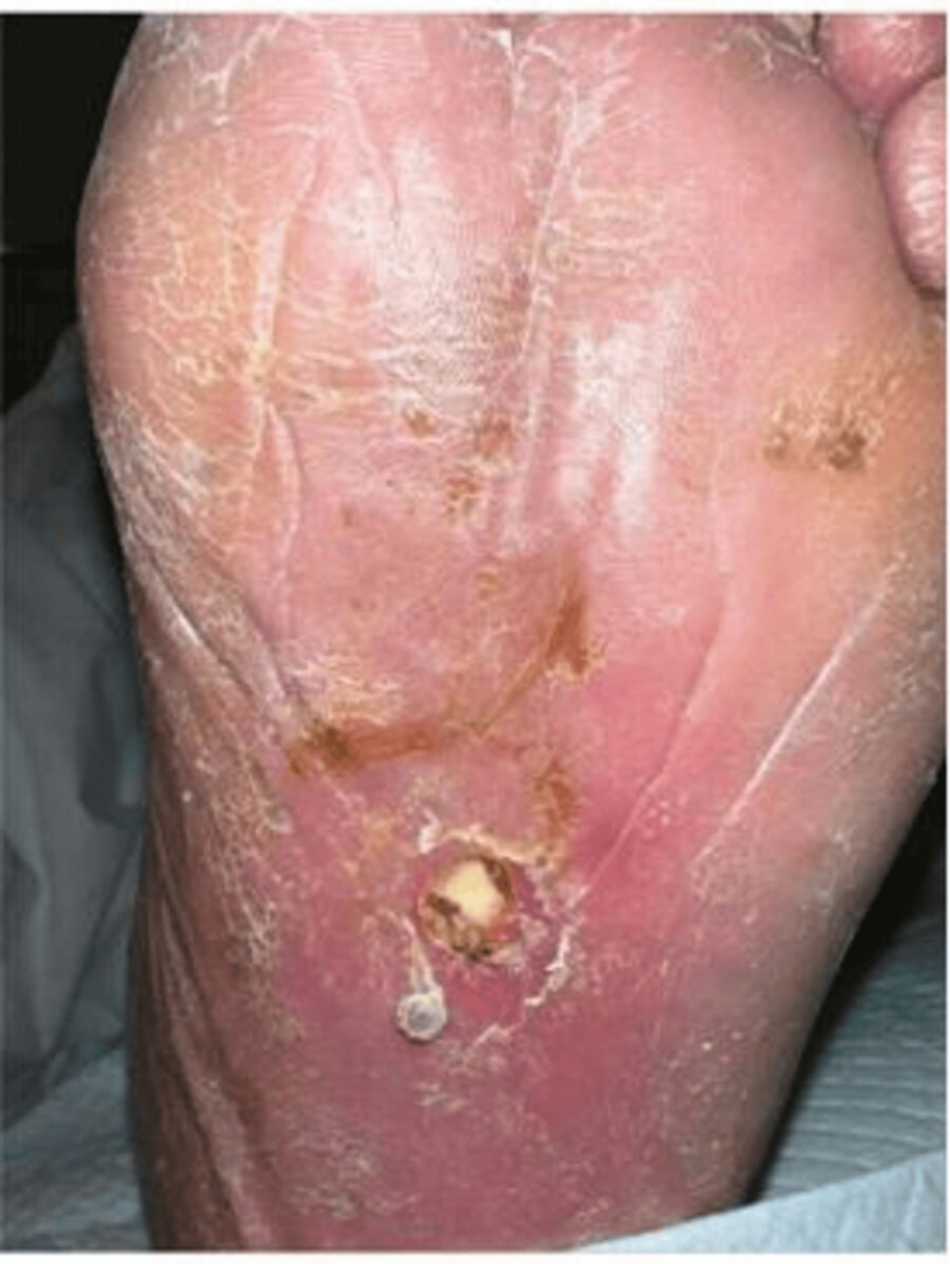
Plantar ulcer. Ulcer identified during examination on the left foot before drainage. Cellulitis is a source of infection.

**Figure 2:**
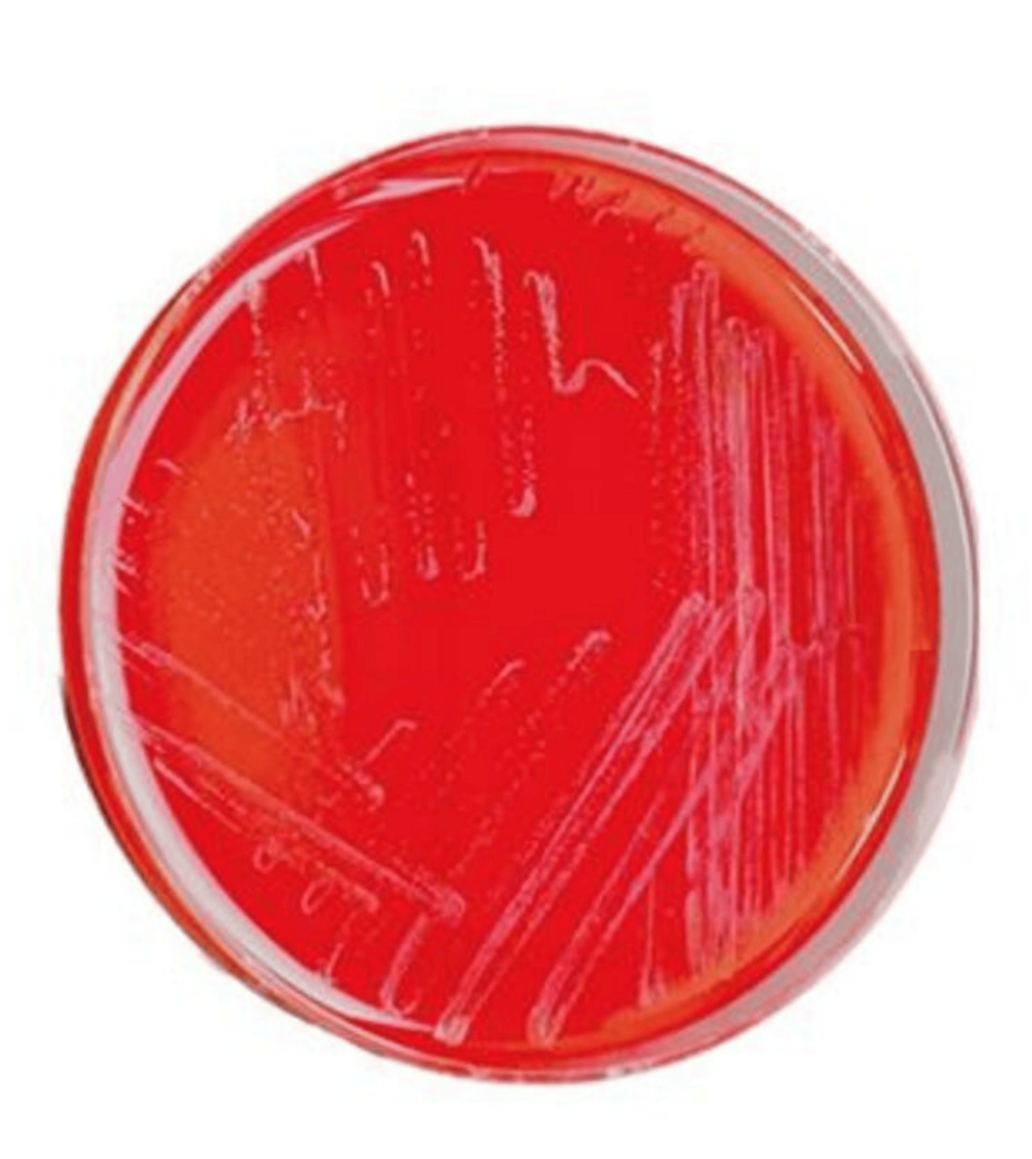
Sheep Blood Agar Plate. T. praeacuta colonies on blood agar captured at 48 hours post-incubation.

Left lower extremity examination was remarkable for erythema of the left foot extending up to the knee. An ulcer was present on the plantar aspect of the left foot, measuring 1 cm x 1 cm x 0.4 cm, with purulent discharge, a fibrotic base, and exposed plantar fascia (Figure 1). A small amount of seropurulent discharge was noted, along with peri-wound erythema and edema surrounding the wound borders. The presentation was consistent with sepsis due to left lower extremity cellulitis. The MRI of the foot was concerning for sinus tract and abscess formation but was negative for osteomyelitis. Debridement of the wound was followed by incision and drainage. The wound culture was positive for *Streptococcus pyogenes*. Anaerobic blood culture was positive post-incubation (Figure 2). Gram staining revealed gram-positive, rod-shaped organisms. Therefore, subcultures of the broth were performed onto blood agar incubated at 37°C, chocolate agar incubated at 37°C in 5% CO_2_, and blood agar incubated in an anaerobic atmosphere for 48 h. Bacterial growth was observed only on the anaerobic plate. Identification of the strain using Rapid ANA (ThermoFisher Scientific, Waltham, MA, USA) was inconclusive. The specimen was sent out for MALDI-TOF, revealing *T. praeacuta* as an offending agent. During the inpatient course, the patient was treated with IV vancomycin, cefepime, and ertapenem. The patient was ultimately discharged to a skilled nursing facility with six weeks of IV ertapenem therapy.

## Discussion

Cellulitis is common in the middle to older population. The incidence of cellulitis is about 200 cases per 100,000 patients per year [7]. The most common cause of cellulitis is the beta-hemolytic streptococci family, most commonly group Streptococcus or *Streptococcus pyogenes* [8,9]. *Staphylococcus aureus*, including methicillin-resistant strains, is a less common cause [9]. Other less common causes of cellulitis include *Haemophilus influenzae* type b, clostridia and non-spore-forming anaerobes, *Streptococcus pneumoniae*, and *Neisseria meningitidis* [10-14]. The clinical significance of identifying correct etiologic agents causing disease and directing targeted antimicrobial therapy cannot be overstated, as appropriate antibiotic treatment helps decrease the duration of hospital stay and prevents the emergence of newer multidrug-resistant organisms [15].

The Tissierella genus is associated with five species: *T. praeacuta*, *T. carlieri*, *T. creatinine*, *T. creatinophila*, and *T. pigra* [3]. Of these, only *T. praeacuta* is known to cause clinically relevant infection in humans [1,2]. *T. praecuta* has been described as a causative organism in brain abscesses [4], hepatic abscesses, pyonephrosis [2], pyometra [5], chronic sacral wounds, decubitus ulcers [3], pseudoarthrosis of the femur [2], osteomyelitis [1], and gas gangrene of the eyelid [6]. 

*T. praeacuta* is now used synonymously with *Clostridium hastiforme* (*C. hastiforme*). Clostridium stains gram-positive, particularly in the early stages of growth, whereas *T. praeacuta* has been described as a gram-negative organism in most case reports previously. Despite this discrepancy in Gram staining, 16S rRNA gene sequences displayed 99.9 % similarity in a study conducted by Farrow et al [16]. Moreover, Gram staining and spore formation are not used as a basis of relatedness [16]. Alternatively, Bae et al. [17] found *T. praeacuta* to be Gram-positive, which was confirmed by a parallel confirmatory KOH test to rule out false negative results using the technique used by Power [18]. DNA-DNA relatedness was estimated at 96.5 %, which was well above the suggested 70% needed to establish strains belonging to the same species. *T. praeacuta* and *C. hastiforme* were also found to be identical based on their biochemical characteristics. It was thus concluded that *C. hastiforme* is a later synonym of *T. praeacuta* [17].

Identification of the organism at the bench requires growth-dependent methods such as culturing colonies on blood agar, subjecting them to aerotolerance testing and gram staining, and biochemical methods such as Rapid ANA II testing and VITEK [17,19,20]. The growth-dependent system of identifying anaerobic organisms, while reliable and convenient, can be a time-consuming process. Of these, gram staining can yield false negative results typically with gram-variable organisms or due to over-decolorizing for prolonged periods or by using old cultures. Rapid ANA is used to identify organisms through biochemical-dependent methods rapidly, but it has limitations, particularly in identifying anaerobes [21]. Rapid ANA is a four-hour kit test that identifies anaerobes by utilizing an assay of preformed enzymes against chromogenic substrates. The biochemical profile generates a six-digit code, which is then interpreted either from the code book or the manufacturer's database. A comparison study by Hussain et al. [21] between the growth-dependent system and Rapid ANA correctly identified only 71% of the clostridia species (an anaerobe). The panels containing the clostridia strains were found to be challenging to interpret.

*T. praeacuta* can be identified by using newer techniques such as MALDI-TOF and mass spectrometry (MS) [3,22]. However, the database needs to contain the mass spectrum of this strain [3]. Molecular techniques such as identification by 16S rRNA sequencing can also be used for inconclusive cases [2]. *T. praeacuta* is known to have antibiotic sensitivity to beta-lactams, chloramphenicol, rifampin, and metronidazole [2,3].

## Conclusions

This is the first documented case report of gram-positive *T. praeacuta* bacteremia in a patient with sepsis resulting from lower extremity cellulitis without concomitant osteomyelitis, confirmed by matrix-assisted laser desorption/ionization-time of flight (MALDI-TOF) mass spectrometry (MS). Although rare, *T. praeacuta* is well characterized and has a complex history of bacterial taxonomy and nomenclature classification, being most recently characterized as synonymous with *C. hastiforme*. Due to many laboratories' policies to limit the workup of gram-negative rods, it is possible that *T. praeacuta* and *C. hastiforme* are under-reported. Additionally, the gram-variable nature of *T. praeacuta* can cause misdiagnosis. Rapid ANA results can be challenging to interpret for anaerobes and may be inconclusive, as was the case in our case. Newer techniques, such as the matrix-assisted laser desorption/ionization-time of flight (MALDI-TOF), can aid in diagnosing rare cases such as *T. praeacuta*, emphasizing the importance of reporting such cases to build the database that contains the mass spectrum of this strain. Molecular testing with 16S rRNA sequencing can also be helpful in successfully identifying this rare organism.
